# Thymectomy for non-thymomatous myasthenia gravis: a propensity score matched study

**DOI:** 10.1186/s13023-014-0214-5

**Published:** 2014-12-24

**Authors:** Carolina Barnett, Hans D Katzberg, Shaf Keshavjee, Vera Bril

**Affiliations:** Division of Neurology - Department of Medicine, University of Toronto and University Health Network, Toronto, Canada; Institute of Health Policy, Management and Evaluation, University of Toronto, Toronto, Canada; Division of Thoracic Surgery, Department of Surgery, University Health Network, Toronto, Canada

**Keywords:** Myasthenia Gravis, Thymectomy, Propensity Score, Bayesian

## Abstract

**Background:**

The efficacy of thymectomy in patients with non-thymomatous Myasthenia Gravis (MG) is still unclear. Main limitations have been variable outcome definitions, lack of a control group and adjustment for confounding.

**Objective:**

To study the efficacy of thymectomy in achieving remission or minimal manifestation (R/MM) status in patients with non-thymomatous MG.

**Methods:**

Patients with generalized MG and minimum follow-up of 6 months were included. Demographic data and treatments were recorded, as well as the MGFA post-intervention status at the last visit. Propensity scores were used to create a matched cohort of treated and untreated patients. Standard and Bayesian Cox models were used to study treatment effects.

**Results:**

Of 395 patients included, 183(46%) had a thymectomy. Thymectomy patients were younger (p < 0.001), with more females (p < 0.001) and more patients in MGFA classes 4–5 at diagnosis (p = 0.01). A matched cohort of thymectomized patients and controls (n = 98) was created. The hazard ratio (HR) for the matched cohort was 1.9 (CI:1.6-2.3), favoring thymectomy. The predicted R/MM rate was 21% in treated and 6% in controls at 5 years (Absolute difference:15%). A Bayesian Cox model for the matched cohort had an estimated probability of thymectomy efficacy (HR > 1) of 96% using a non-informative prior, and 79% using a skeptical prior.

**Discussion:**

When controlling for potential confounders, thymectomized patients had a higher probability of achieving R/MM status through time compared to controls. This study provides class III evidence of the efficacy of thymectomy in non-thymomatous myasthenia gravis.

## Background

The efficacy of thymectomy in improving outcomes in patients with non-thymomatous myasthenia gravis (MG) is still under study, despite the fact that it has been used in clinical practice for over 60 years [1-3]. The evidence of its efficacy has been based on observational studies with different methodological considerations. For example, different definitions of remission have been used and there has been inconsistent control for confounders. In some cases, relative risks have been used, when time-to-event analyses are more appropriate given different follow-up times [4-6]. These issues were raised in a practice parameter from the American Academy of Neurology in 2000 [7], that reported a systematic review of the literature and found that studies controlling for different confounders, showed conflicting results. The authors concluded that the evidence supported the use of thymectomy as an option to improve outcomes, but that further studies and ideally randomized control trials should be pursued.

In the past years, several case series of thymectomy have been published, and different surgical techniques have been studied, but most of those studies have lacked a control arm and have variable adjustment for confounders [8-11]. Even though the surgical management of MG patients has improved with time and the associated morbidity and mortality are low, especially with less invasive techniques [7,8], thymectomy still conveys risks and associated costs. Therefore, it is imperative to better understand its effectiveness in improving outcomes in these patients.

A recent Cochrane review [12] concluded that there is lack of evidence to support the use of thymectomy in non-thymomatous MG, and that randomized and quasi-randomized studies are needed. A randomized control trial is underway (NCT00294658) [13], but its results are yet not available. The difficulties in performing such a trial create the need for well-designed observational studies to add to the evidence base on this relevant question.

In our center, thymectomy for non-thymomatous MG, is usually performed in patients with generalized MG who are young, and therefore most frequently women. Given this clear bias by indication, where baseline characteristics and associated medical treatments can affect the outcomes, we used propensity scores to create a matched dataset, thus simulating a randomized study. The primary objective of this study was to estimate the treatment effect of thymectomy in achieving remission or minimal manifestation status [as defined by the Myasthenia Gravis Foundation of America (MGFA) classification] in patients with non-thymomatous MG. We hypothesized that thymectomized patients would have a higher probability of achieving remission or minimal manifestation status through time compared to controls.

## Methods

### Data collection

Records from consecutive MG patients who attended the Neuromuscular Clinic, Toronto General Hospital, from January 2000 to August 2013, were retrospectively reviewed. Inclusion criteria were: confirmed generalized MG and minimal follow-up of 6 months. We excluded patients with purely ocular disease, thymoma or missing data on occurrence of thymectomy and/or post-intervention status. The diagnosis of MG was based on the clinical presentation and abnormal antibody status (acetylcholine or muscle specific kinase) or single fiber electromyography. One assessor collected the demographic data, including: age at onset, time to diagnosis, antibody status, medications, thymectomy status (including type of thymectomy) and total time of follow-up. The Myasthenia Gravis Foundation of America (MGFA) class at diagnosis and MGFA post-intervention status (PIS) [14] at the last visit, were recorded separately by a neuromuscular physician (CB). Following the MGFA-PIS classification, remission was defined as a minimum of 1 year without symptoms (eye closure weakness accepted) and without pyridostigmine. Asymptomatic patients for at least a year but using pyridostigmine or patients with minimal signs or symptoms (i.e. isolated ptosis) for at least a year were classified as minimal manifestations. Given the difficulties of timing relapses retrospectively, these were not considered, and only the clinical status at the last visit was used. The primary outcome was the presence of remission or minimal manifestation status (R/MM) according to the MGFA-PIS at the last visit. The University Health Network Research Ethics Board approved this study.

### Statistical analyses

1. *Descriptive statistics and missing data:* Continuous variables were expressed by means and SD, and nominal variables by number of observations and proportions. Variables with < 20% missing data were dealt by with multiple imputation. Variables with > 20% missing data were not included in the models. All analyses were done with R-statistical software (The R foundation for statistical software, version 3.0.2).
2. *Propensity Score (PS) Models:* Several models were tested, and the model with the best balance between the variables was chosen [15]. All models tested included the main drivers of the decision to operate in our center: age and sex. Since these are related, they were modeled as an interaction. In addition, several other potential confounders were tested, alone and in combination, since the incorporation of other confounders has shown to improve estimates and balance of PS models [15]. The variables tested in the models were: time to diagnosis, MGFA status at diagnosis (I,II,III, and IV-V), and use of prednisone, azathioprine and mycophenolate mofetil during the follow-up period. Other medications such as methotrexate, cyclosporine and rituximab are infrequently used and were not included in the PS models, but their balance was tested before and after matching. IVIG and plasma exchange (PLEX) are used for worsening disease or crisis, and also pre-thymectomy, but are not routinely used as chronic treatment. Therefore, as they are not expected to affect the likelihood of achieving R/MM status, we did not include them in the models. However, we described the proportion of patients receiving these treatments during follow-up in the matched cohort.
3. *Matching and Balance of the Covariables*: Matching was done in a 1:1 proportion without replacement, matching on the PS using a nearest-neighbor algorithm with a caliper of 0.2 [16]. This was done using the MatchIt package for R [17]. To test for balance of the different variables on the matched dataset, we used the absolute standardized difference (ASD), where values < 0.1 are considered indicators of appropriate balance. This approach is preferred to the use of significance testing, since p-values are dependent on sample size and in smaller samples might not be sensitive enough to detect imbalance [16]. All subsequent analyses on the matched dataset used frailties or random effects to account for the paired nature of the data [18].
4. *Time-to-event Analyses:* The primary outcome was the hazard ratio (HR) for achieving R/MM status at the last visit, fitting a Cox model on the matched dataset. Patients who were not in R/MM at the last visit were right-censored. Using the survival models coefficients, we estimated the probability of achieving R/MM status at 5 years. That time frame was chosen to detect longer-term effects of treatment, and was below the mean follow up time ranges for both groups. As a secondary analysis, a Cox model was built for the complete, unmatched dataset, adjusting for all the possible confounders, including age, sex, medications, time to diagnosis and MGFA class at baseline. For the secondary outcome, the use of prednisone at the last visit was modeled in a time-to-event fashion, whereby the hazard ratio for being on prednisone (HR) was estimated on the matched dataset. For all Cox models, the proportional hazard assumption was tested through the Schoenfield residuals [19].
5. *Bayesian Models:* To estimate the probability of thymectomy effectiveness (probability of HR > 1), Bayesian proportional hazard models were built for the matched and unmatched datasets. For each dataset, we used different prior probabilities as a form of sensitivity analysis [20]. First, we used non-informative priors, assuming no previous knowledge of the role of thymectomy in this population. This prior had a normal distribution with a log HR mean of 0 and precision of 10^−6^. We then used a skeptical prior, assuming a prior probability of no effect of thymectomy (HR = 1, neither beneficial nor harmful) with a 95% CI between 0.6 and 1.4. This last model placed more strength in the prior probability of no thymectomy effect, therefore giving more strength to the null hypothesis [21,22]. For all models, we assumed a constant hazard rate and therefore we used an exponential distribution. We used Monte Carlo Markov Chain (MCMC) simulations with Gibbs sampling, using JAGS (v.3.0.2) [23] through the rjags package for R [24]. All models were initiated with 3 chains. To ensure convergence, 10,000 burn-in iterations were done, followed by 10,000 samples to calculate the estimates. Convergence was tested by visualizing the traces from the 3 chains and by the Gelman-Rubin statistic. The parameters calculated were the median estimated HR with 95% Credible Intervals (CrI), the probability of a HR >1 (in favor of thymectomy), the median probabilities of achieving R/MM status at 5 years and the median absolute difference in R/MM status between groups at 5 years.

## Results

### Patients

395 patients met the inclusion criteria. Of these, 183 (46%) had had a thymectomy. Thymectomized patents were younger, and had a higher proportion of women (p < 0.001). They also had longer mean follow-up times and more patients were in MGFA classes IV-V at diagnosis. Detailed characteristics of both groups are shown in Table 1. At the last visit, 40(22%) had R/MM status in the thymectomy and 49(23%) in the control group. In the thymectomized group, data on the operative protocol were available for 125 patients. Of these, 104 (83%) had a trans-cervical video assisted thymectomy, 20 (16%) a trans-sternal thymectomy and 1(1%) both. There were missing data on time to diagnosis (14.5%) and baseline MGFA (3%). Data on acetylcholine receptor antibodies were available for 45% of patients, and thus antibody status was not included in the models. There were no missing data for the other variables included in the PS models.

**Table 1:** **Demographic characteristics of matched and unmatched cohorts**

**Characteristics**	**Unmatched (n = 395)**			**Matched (n = 98)**		
	**Thymectomy (n = 183)**	**Controls (n = 212)**	**Absolute standardized difference**	**Thymectomy (n = 49)**	**Controls (n = 49)**	**Absolute standardized difference**
Age (mean ± SD)	34.8 ± 14.1	63.7 ± 12.7*	2.33^†^	49.8 ± 14.3	50.1 ± 13.2	0.02
Females [n (%)]	124(68)	89(42)*	2.03^†^	25(51)	27(50)	0.08
Time to Dx (mean ± SD)	14.8 ± 26.1	16.9 ± 31.8	0.55^†^	17.8 ± 25.6	18.3 ± 27.3	0.02
MGFA class at diagnosis n [(%)]						
MGFA I	16(9)	31(15)*	0.21^†^	5(10)	4(8)	0.07
MGFA II	57(31)	81(38)	0.15^†^	21(43)	23(47)	0.08
MGFA III	70(38)	73(34)	0.08	17(35)	15(31)	0.08
MGFA IV/V	40(22)	27(13)*	0.22^†^	6(12)	7(14)	0.05
Medications [n(%)]						
Prednisone	130(71)	166(78)	0.16^†^	35(71)	33(67)	0.09
Azathioprine	109(60)	118(56)	0.08	30(61)	31(63)	0.04
Mycophenolate	32(18)	41(19)	0.05	6(12)	7(14)	0.05
Other immunosupressants	28(15)	21(10)	0.15	4(8)	5(10)	0.07
Follow-up (mean ± SD)	118.9 ± 115.9	67.4 ± 52.8*	0.44^†^	95.9 ± 103.1	76.4 ± 54.6	0.16^†^

Continuous data are expressed as median and range.

Nominal data are expressed as number and proportion of patients.

MGFA: Myasthenia Gravis Foundation of America. The proportion of patients in each MGFA class at diagnosis, before treatment, is presented for both groups.

Time to Diagnosis (Dx) and Follow-up are in months.

Other immunosupressants include: methotrexate, rituximab, cyclosporine and cyclophosphamide.

^†^Absolute Standardized Difference > 0.1, indicating poor balance of the variable between groups.

*p < 0.05. p-values are less sensitive than the Absolute Standardized Difference to detect imbalance.

The matched dataset has excellent balance of all the variables, except mild residual imbalance of follow-up times. This was accounted with time-to-event analyses.

### PS matching

The final PS model included the interaction between age and sex, and also time to diagnosis and MGFA class at diagnosis. This resulted in a matched dataset with n = 98. This dataset demonstrated adequate balance of all the covariables (standardized difference <0.1), with only residual imbalance in the time of follow up (ASD = 0.16). Table 1 shows the characteristics of the matched dataset compared to the total cohort. Excluding pre-surgical treatments, PLEX was given to 12(24%) and 14(28%) of thymectomy patients and controls respectively (p = ns) during follow-up. 17(35%) patients received IVIG in the thymectomy and 23(47%) in the control group (p = ns).

### Cox models

**Figure 1:**
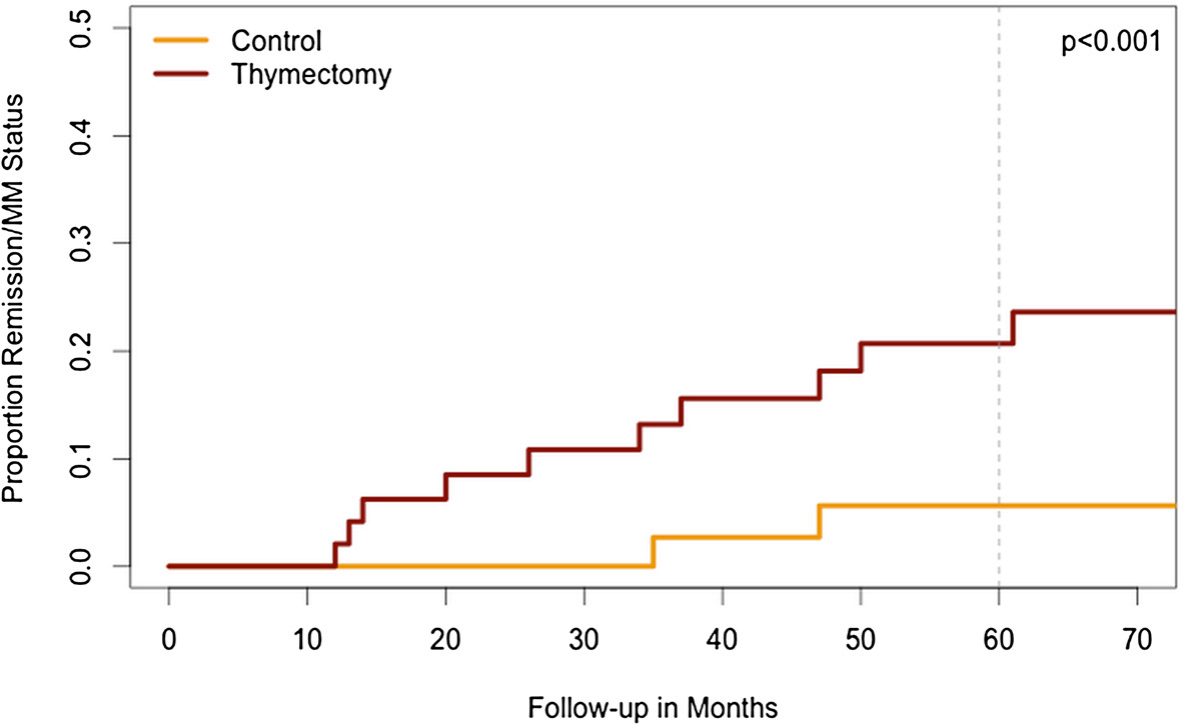
**Cumulative proportion of remission/MM status in thymectomy and controls, in the matched dataset.** Patients who had thymectomy had a higher likelihood of achieving Remission/MM status through time, compared to controls. (HR:1.9, CI:1.6, 2.3. p < 0.001). The absolute estimated difference at 5 years (60 months) was 15%.

**Figure 2:**
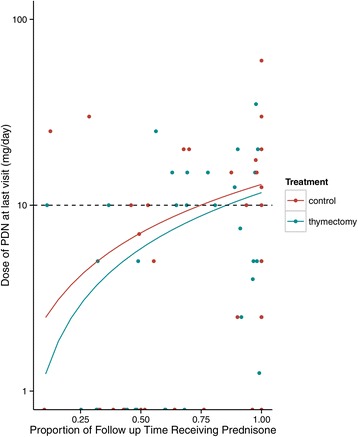
**Relationship between prednisone treatment duration and dose of prednisone on the last visit in the matched cohort.** The overall use of prednisone through time is presented as a ratio of the time under prednisone in months, over the total follow-up time, to account for different follow times. Patients with higher ratios of prednisone use through time were more likely to have higher doses at the last visit (r = 0.5, p < 0.001) for both groups. There was no significant difference between groups, although controls tended to have higher doses of prednisone at the last visit (p = ns).

In the matched dataset, thymectomized patients had a higher likelihood of achieving R/MM with time than the controls (HR: 1.9, CI:1.6, 2.3). In the unmatched dataset, the adjusted estimated HR was similar, but it did not reach statistical significance (HR: 1.5, CI:0.8, 2.8). Using the matched data, the estimated rates of R/MM at 5 years were 21% (CI:16, 40) for the thymectomy group and 6%(CI:0–13)for the controls. This yields an absolute difference of 15% (CI:1, 29), with a NNT = 7. Details of these results are in Table 2, and Figure 1 represents the cumulative hazards for R/MM status in the matched data. Regarding the use of prednisone at the last visit, the thymectomy group was less likely to be on prednisone than controls (HR: 0.8, CI:0.7-0.95, p = 0.005). At 5 years, the predicted proportion of patients using prednisone was 23% in thymectomy patients and 30% in controls. Figure 2 depicts the relationship between the proportion of follow-up time using prednisone and the dose of prednisone on the last visit for treated and controls.

**Table 2:** **Hazard ratios, absolute differences for matched and unmatched dataset**

	**HR for R/MM status**	**Absolute difference in R/MM at 5 years**	**Probability HR > 1**
**Standard Cox models**			
Unmatched data	1.5 (CI: 0.8, 2.8)	−3% (CI: −10, 4)	--
Matched data	1.9 (CI: 1.6, 2.3)*	15% (CI: 1, 29)	--
**Bayesian models**			
Matched data, uninformative prior	2.2 (CrI: 0.9, 6.0)	9% (CrI: −1, 27)	96%
Matched data, skeptical prior	1.2 (CrI: 0.8, 1.6)	2% (CrI: −1, 9)	79%
Unmatched data, uninformative prior	1.9 (CrI: 1.0, 3.3)	20% (CrI: 2, 38)	98%
Unmatched data, skeptical prior	1.2 (CrI: 0.8, 1.7)	4% (CrI: −3, 12)	86%

Values for the standard Cox models are expressed with a 95% confidence interval (CI).

The Bayesian models used 10000 iterations. The values presented are the median and 95% credible intervals (CrI).

HR: Hazard Ratio.

HR > 1 indicates increased likelihood of achieving R/MM, favoring thymectomy.

R/MM: Remission or Minimal Manifestation Status.

*p < 0.0001. p values are not part of Bayesian analyses.

### Bayesian models

**Figure 3:**
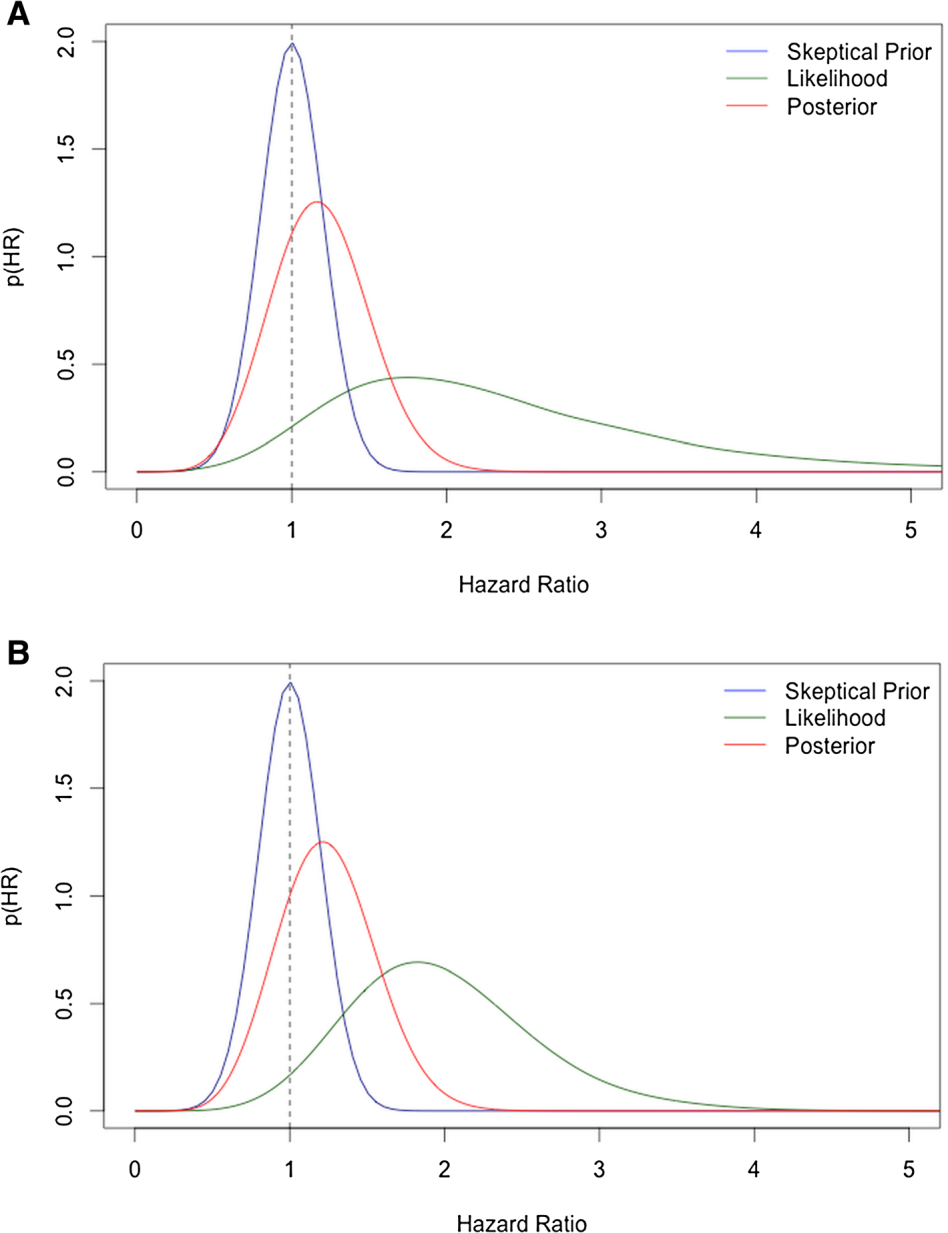
**Bayesian tri-plots of the efficacy of thymectomy using different prior probabilities in the matched and unmatched datasets.** In Figure 3 **A** and **B**, the blue line depicts the skeptical prior, assuming prior belief of no efficacy of thymectomy, with a mean HR = 1 and 95% CI:0.6 -1.4. The green line depicts the likelihood, which is the probability of thymectomy efficacy for each model, using the observed data only (uninformative prior). The red line reflects the posterior probability, which incorporates the prior belief to the likelihood. Figure 3 **A** shows the probabilities for the matched dataset. The likelihood has a 96% probability of thymectomy efficacy (HR >1), with a posterior probability of 79% after incorporating the skeptical prior. Figure 3 **B** reflects the unmatched dataset. The likelihood has a 98% probability of thymectomy efficacy, with a posterior probability of 86% after incorporating the skeptical prior.

Using an uninformative prior, the unmatched data had a median HR for achieving R/MM status of 1.9 (CrI: 1.0, 3.3), with a 98% probability of having a HR > 1 (favoring thymectomy); in the matched data, the median HR was 2.2 (CrI:0.9, 6.0), with a probability of thymectomy efficacy of 96%. Using a skeptical prior, the median HR was 1.2 (CrI:0.8, 1.6), with a probability of thymectomy efficacy of 79% in the matched data, and 1.2 (CrI: 0.8, 1.7), with a probability of thymectomy efficacy of 86% in the unmatched data. Table 2 shows the parameters of the different Bayesian models, and Figure 3 Shows Bayesian tri-plots, illustrating the different probabilities (prior, likelihood and posterior) of thymectomy efficacy in the matched and unmatched data.

## Discussion

In this study, we used novel methods to assess the treatment effect of thymectomy in non-thymomatous myasthenia gravis. The use of propensity scores is widespread in the literature [25], but to our knowledge, it has not been used before for this clinical question. Further, we incorporated Bayesian analyses to assess the overall probability of thymectomy efficacy, which can be easier to interpret from a clinical perspective. Bayesian methods allow for incorporating previous knowledge or beliefs. Since previous data on thymectomy efficacy are inconsistent, we used 2 approaches to estimate our prior possibilities: assuming no knowledge (non-informative prior) and assuming evidence of no efficacy (skeptical prior). The first assumption allowed us to place more emphasis in our observed data, and the second assumption served to incorporate the “worst case scenario” for thymectomy, giving more weight to a previous belief of no efficacy [26]. Finding an effect in this skeptical model strengthens our findings. In addition, Bayesian methods are not limited by sample size, and that is an asset when studying rare diseases [27] such as MG, and in this particular case of a matched dataset with a smaller sample size.

We found that thymectomy is associated with a high probability of achieving remission or minimal manifestation status, when compared to no surgery in patients with non-thymomatous MG after controlling for several confounders, including age at onset, time to diagnosis, MGFA class at diagnosis and the use of several immunosupressants. The different times of follow-up were accounted for using a time-to event approach. Therefore, our study follows the recommendations by the AAN practice parameter [7] and the recommendations for MG research issued by the MGFA [28]. For robustness, we used different models to assess the outcome, including standard and Bayesian models in both the matched and unmatched datasets, and the findings were consistent across models and datasets. Even when using a skeptical model, the probability of efficacy remained high. The absolute difference estimates for R/MM rates at 5 years between treatment groups ranged between 2% in the skeptical Bayesian model with matched data to 20% in the uninformative Bayesian model with unmatched data. A relevant question that arises from these findings is how big should the treatment effect be (i.e. absolute difference) to be significant from a clinical and cost-effective perspective. Future cost-effectiveness studies incorporating the results from the ongoing RCT will be needed to answer that question. In addition, as secondary outcome, we found that thymectomy was also associated with a higher likelihood of being free from prednisone through time. This is of relevance, given the known secondary side effects of long-term steroid use, and the documented correlation between prednisone use (especially high dose) and reduced quality of life in MG patients [29].

We found a low rate of remission (10%) compared to previously published thymectomy studies, and therefore, we included minimal manifestation status in the primary outcome for statistical considerations. Minimal manifestation includes patients who have been asymptomatic for at least a year but are using pyridostigmine, as well as patients with minimal symptoms such as isolated ptosis. Therefore, we considered it as a good clinical outcome. The relatively low rate of remission might be explained by several factors: firstly, we used the MGFA-PIS classification, that requires at least 1 year without symptoms to classify as remission. Previously published studies assessing thymectomy have used variable definitions of remission, with variable lengths of time, and this might explain some of the differences. Secondly, our data is from a single, academic referral center. It is possible that milder cases are being followed at community hospitals, possibly translating into a cohort of more severely affected patients in this study. Therefore, our findings can only be generalized to similar clinical settings. Additionally, we excluded patients with very short follow-up times. This is because our aim was to look at the longer-term effect of thymectomy. Even though there are some reports of early improvement after surgery, an effect of pre-surgical IVIG or PLEX can’t be ruled out; therefore we excluded those cases from our cohort.

The use of observational data has advantages and limitations. Observational studies are typically more feasible than RCTs, which are costly and hard to implement for several reasons, and so far, only one RCT addressing this question is on course. RCTs provide some of the strongest evidence by balancing confounder factors by default and prospectively collecting data. However, the inclusion criteria are usually more restrictive, affecting the generalizability of the results. In that sense, observational data can be more pragmatic, because they can better represent the “real world” of patients with different characteristics and treatments that often make them not eligible for RCTs. However, confounding is always a concern in observational studies, therefore the need for rigorous data collection and analysis. We eagerly await the results of the ongoing RCT, but we believe that further studies on prospectively collected observational data are also required, since it is unlikely that another RCT addressing this question will be carried in the future.

This study is not without limitations. Propensity scores can only adjust for the known variables, as opposed to RCTs were the unknown variables are assumed to be balanced by the randomization. In our study, the main unknown is the Acetylcholine receptor antibody status, as this test is not covered by the Ontario Health Insurance Plan, and thus, many patients with a confirmed MG diagnosis (based on clinical and electrodiagnostic criteria) are not tested. Therefore, our findings might be different to a population of only seropositive patients, who could in theory have better response to thymectomy [30]. In addition, most of our patients underwent transcervical video assisted thymectomy, and it is possible that the treatment estimates differ from other surgical techniques. The transcervical procedure is less invasive and some authors consider that less invasive techniques might result in residual thymic tissue and therefore might be less effective than trans-sternal surgery [3]. However, we still found a positive effect with a predominance of transcervical thymectomy, supporting its use. A matched study comparing different surgical approaches can answer whether there is different efficacy with different surgical techniques, and also compare complications and associated costs.

The retrospective data collection is another potential source of bias. Even though data on the use of immunosupressants were available for all patients as a dichotomous variable (yes/no), we could not capture the different doses through time, which could influence the outcome. We tried to maintain blinding by having different assessors capture the demographic and the outcome data. However, given the retrospective review, blinding was not always maintained and that could have introduced bias. A prospective study, with dynamic collection of medications and doses, could provide more accurate treatment effects, modeling the immunosuppressant data as time-dependent covariables, and ensuring blinding assessment of outcomes. In the case of prednisone, prospectively collecting data can also allow for estimating the cumulative use of prednisone through time, which can be an important outcome. Additionally, a prospective study could look into the relapse rate in patients achieving remission, which we did not include in this study.

## Conclusion

In summary, using novel statistical techniques in observational data, we found that thymectomy is associated with a high probability of achieving remission or minimal manifestation status and of being free from prednisone when compared to controls. Further prospective studies are needed, and if the evidence of thymectomy benefit holds, cost-effectiveness studies will be important in the future to increase our understanding of its role in the management of MG patients.
